# Enhancement of CO_2_ adsorption on activated carbons produced from avocado seeds by combined solvothermal carbonization and thermal KOH activation

**DOI:** 10.1007/s11356-023-28638-y

**Published:** 2023-07-13

**Authors:** Joanna Siemak, Beata Michalkiewicz

**Affiliations:** Faculty of Chemical Technology and Engineering, Department of Catalytic and Sorbent Materials Engineering, West Pomeranian University of Technology in Szczecin, Piastów Ave. 42, 71-065 Szczecin, Poland

**Keywords:** CO_2_ adsorption, Carbon capture, Avocado seeds, Ultramicroporous activated carbons, Selectivity, Solvothermal carbonization

## Abstract

A new strategy for ultramicroporous activated carbons production from avocado seeds was developed. Combined solvothermal carbonization and thermal KOH activation were conducted. Solvothermal carbonizations were performed in a stainless-steel autoclave lined with Teflon at the temperature of 180 °C for 12 h in three different liquids (water, methanol, isopropyl alcohol). Chars were activated by KOH. The carbonization combined with activation took place in the oven at 850 °C for 1 h. All the samples were very good CO_2_ sorbents. The highest CO_2_ adsorption at a pressure of 1 bar was achieved for activated carbon produced using isopropanol. The best carbon dioxide adsorption was equal to 6.47 mmol/g at 0 °C and 4.35 mmol/g at 20 °C.

## Introduction

The emissions of anthropogenic CO_2_ have caused a significant impact on the global climate (Zhang et al. 2019). CO_2_ is mainly produced by fossil fuel combustion but also accompanies cement production, petrochemical, and other chemical processes (Huang et al. 2022). Activated carbons produced from waste biomass are auspicious and low-cost materials for CO_2_ adsorption (Creamer et al. 2014).

Some methods of activated carbons production from avocado seeds have been described in the literature. Avocado seeds were applied for activated carbon production for phenol removal from water (Rodrigues et al. 2011). The carbonization was performed in an oven at 800^o^C and then activated with CO_2_ at 900 °C. Carbon material with a low specific surface area (206 m^2^/g) and a negligible volume of mesopores (0.048 cm^3^/g) and micropores (0.052 cm^3^/g) were obtained.

The procedure of activated carbon synthesis in a microwave oven using ZnCl_2_ as an activator was described (Leite et al. 2017). A material with a relatively high specific surface area (1432 m^2^/g) and a low volume of mesopores (0.325 cm^3^/g) and micropores (0.119 cm^3^/g) were obtained. Such properties allowed this material to be used as a sorbent of resorcinol and 3-aminophenol from aqueous solutions.

The procedure of activated carbons production from avocado seeds by pyrolysis at the temperature range 500–700 °C and activation by ZnCl_2_ was presented (Leite et al. 2018). Materials with a relatively high specific surface area (1122–1584 m^2^/g), medium mesopore volume (0.475–0.691 cm^3^/g), and low micropore volume (0.084–0.156 cm^3^/g) were obtained. Such properties allowed the use of these activated carbons as sorbents for amoxicillin, caffeine, captopril, enalapril, and meloxicam.

As an activating agent in the synthesis of activated carbons from avocado seeds, sulfuric acid was used at a temperature of 100 °C (Bhaumik et al. 2014). A material with a low specific surface area (14 m^2^/g) and a low pore volume 0.0323 cm^3^/g was received. This material was used for the adsorption of Cr(VI) ions from aqueous solutions.

The method of activated carbons production based on activation with H_3_PO_4_ at the temperature range 800–1000 °C was described (Elizalde-González et al. 2007). The material with the highest blue 41 dye adsorption had a low surface area (143 m^2^/g) and a low pore volume 0.073 cm^3^/g.

The production of activated carbons from avocado seeds by carbonization in nitrogen or carbon dioxide at 600–1000 °C was presented (Salomón-Negrete et al. 2018). The obtained materials exhibited low specific surface area (52–300 m^2^/g), low pore volume (0.051–0.172 cm^3^/g), and especially micropores (0.019–0.122 cm^3^/g). Such properties allowed using these activated carbons as sorbents of fluorine ions from aqueous solutions.

To the best of our knowledge, avocado seed as a source of activated carbon has been described, as of today, only in six publications listed above. The above review of the literature clearly showed that so far, it has not been possible to develop a method of activated carbons production from avocado seeds with high microporosity, which is essential for sorbents with high CO_2_ adsorption.

In this work, we reported for the first time avocado seeds as carbon precursors for CO_2_ sorbents. A new strategy for ultramicroporous activated carbons production from avocado seeds was developed. We demonstrate that by a combination of solvothermal carbonization and thermal KOH activation allowed to produce activated carbons with uniform ultramicropores (∼ 0.50 nm) and with the enhancement of CO_2_ adsorption. Under 1 bar, the CO_2_ adsorption at a temperature of 0 °C ranged from 6.47 to 6.31 mmol/g and at 20 °C from 4.13 to 4.13 mmol/g. According to our knowledge, these values are very high.

## Materials and methods

### Materials

The carbon precursor, avocado seeds, were bought from supermarkets in Poland. The following reagents were purchased from Chempur (Piekary Śląskie, Poland): methanol, KOH, HCl 35–38%. Isopropyl alcohol was provided by P.P.H. Stanlab Sp. Z o. o. (Lublin, Poland). All chemicals mentioned above were of analytical grade.

Nitrogen (99.999% purity) and carbon dioxide (99.999% purity), obtained from Messer Polska Sp. z o. o., were used for adsorption and samples characterization.

### Activated carbon synthesis

The dried avocado seeds were fine powdered. Solvothermal carbonizations of avocado seed in three different liquids (water, methanol, isopropyl alcohol ) were performed in a stainless-steel autoclave lined with Teflon at the temperature of 180 °C for 12 h. The resulting chars were washed with deionized water and dried at 190 °C.

The char was mixed with saturated KOH solution. The mass ratio of char/pure KOH was 1:1. The mixture was left for 3 h and then dried at 190 °C.

The dried mixtures were placed in a tubular furnace and heated to 850 °C under nitrogen flow. The carbonization combined with activation took place in the oven for 1 h. Then, samples were washed with deionized water until neutral pH was achieved. In the end, samples were dried at 190 °C.

### Characterization of the material

To obtain textural properties, nitrogen sorption isotherms at 77 K were investigated. The relative pressure from 9 × 10^−8^ 8 to 0.99 were acquired using a sorption analyzer ASAP 2020 (Micromeritics). Brunauer–Emmett–Teller equation was used to determine the specific surface area (S_BET_). From 5 to 10 adsorption points from 0.001 to 0.01 p/p_0_ were utilized to calculate S_BET_. Total pore volume was calculated on the basis of the nitrogen volume adsorbed at the relative pressure of 0.99. Micropore volume and pore size distribution were analyzed by DFT method.

The X-ray diffraction (XRD) patterns were collected on a X’Pert–PRO, Panalytical, Almelo X-ray diffractometer with 2 θ from 10 to 100°.

The field emission scanning electron microscopy (FE-SEM) images were taken by a SU8020 Ultra-High Resolution Field Emission Scanning Electron Microscope; Hitachi Ltd, under 5 kV voltage.

A sorption analyzer ASAP 2020 (Micromeritics) was applied to measure volumetric nitrogen and carbon dioxide uptake in a pressure range 0.02–1 bar, at a temperature of 0 and 20 °C.

## Results and discussion

**Fig. 1:**
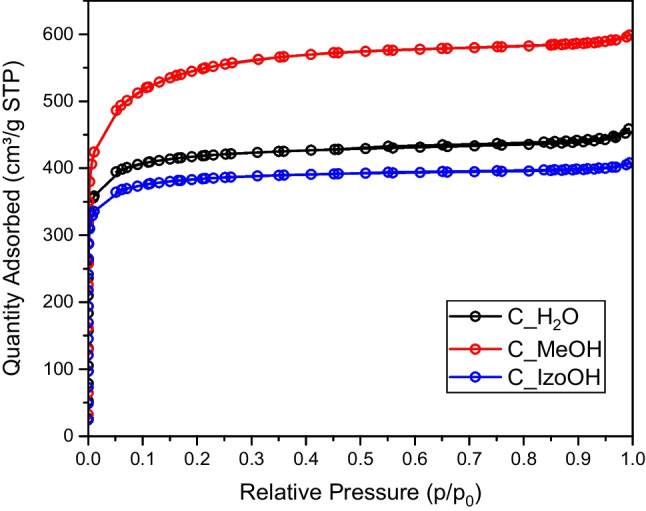
Nitrogen sorption isotherms for activated carbons produced from avocado seeds using various liquids

The nitrogen sorption isotherms investigated at a temperature of 77 K of activated carbons produced from avocado seeds using various liquids are presented in Fig. 1.

On the basis of the isotherm shape illustrating the amount of adsorbed gas at a particular specified pressure, it is possible to define the porosity characteristics of the adsorbent surface (Rashidi et al. 2016). Isotherms can be classified as type I (Sing 1985). As enhanced adsorption at relatively low pressure (less than 0.1 bar) is evident, suggesting that the resulting carbons have a well-developed microporous structure. Moreover, it is visible that a further part of the isotherm reached a plateau that is horizontally aligned with the axis of relative pressure, which suggests that in the structure prevails microporosity (Thommes et al. 2015). The isotherm of C_MeOH was the highest and started from about 400 cm^3^/g. That means that this activated carbon was the most microporous.

Table 1 compiles textural properties of activated carbons from avocado seeds. It was stated that all the materials were highly porous. The specific surface area ranged from 1464 to 2024 m^2^/g, depending on the liquid used. The highest value was observed for methanol. The highest pore volume, 0.9262 cm^3^/g, and micropore volume, 0.6813 cm^3^/g, were achieved for methanol, and the lowest data occurred for isopropanol (V_tot_=0.631, V_micro_=0.506). The micropore content is very high for all the activated carbons. The highest micropore percentages were achieved for C_IzoOH. The values in Table 1 were in good agreement with Fig. 1.

**Table 1:** The textural properties of activated carbons produced from avocado seeds using various liquids

AC	S_BET_	V_tot_	V_micro_	V_tot/_V_micro_
	(m^2^/g)	(cm^3^/g)	(cm^3^/g)	(%)
C_H2O	1590	0.709	0.549	77.41
C_MeOH	2024	0.926	0.681	73.5
C_IzoOH	1464	0.631	0.506	80.2

**Fig. 2:**
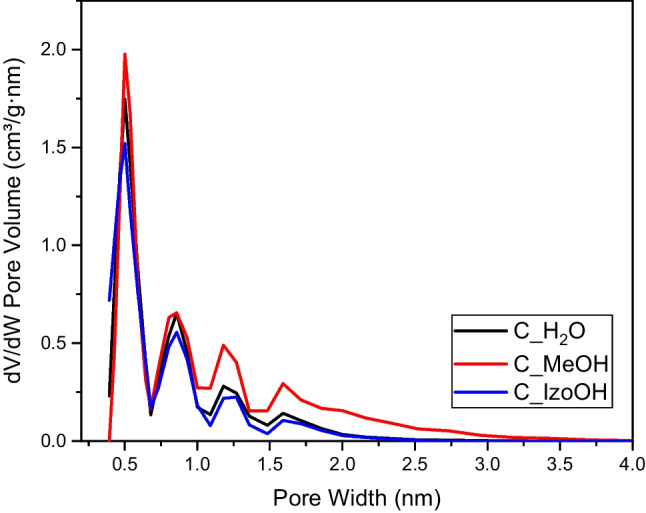
Pore size distribution for activated carbons produced from avocado seeds using various liquids

Figure 2 presents the distribution of pore size calculated by the DFT method based on N_2_ adsorption measured at 77 K. All the activated carbon exhibited four sharp peaks at 0.50, 0.86, 1.19, and 1.60 nm. The first one is the sharpest and the highest, indicating high content of ultamicropores that play an essential role in CO_2_ adsorption (Deng et al. 2015; Serafin et al. 2017). C_H2O and C_IzoOH contained mostly micropores. For C_MeOH, mesopores in the 2–3 nm range were observed. Table 1 also showed that the C_MeOH contained more mesopores than the other activated carbons.

**Fig. 3:**
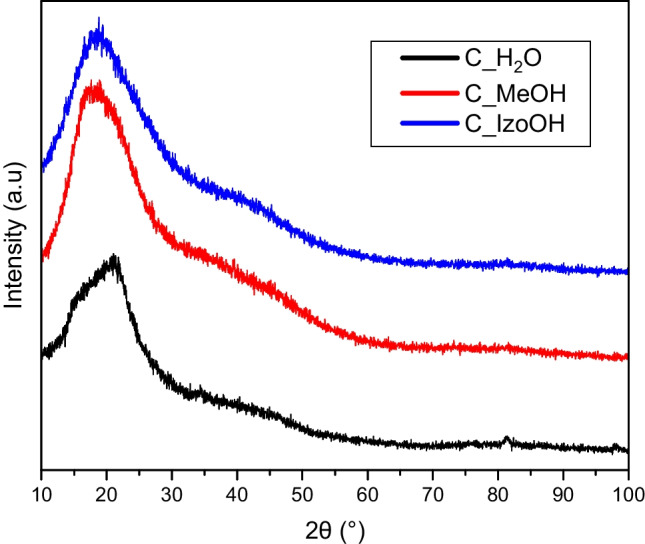
XRD pattern for activated carbons produced from avocado seeds using various liquids

The carbon state of activated carbons was investigated by XRD method (Fig. 3). A very broad, deformed peak between 18 and 21° was observed. This signal can be attributed to the (002) surface of the turbostratic carbon (Wang et al. 2016). The peak of about 44° (100/101) was not visible, confirming the longitudinal dimension, the so-called aromatic sheets, of all the activated carbons were tiny, and the materials were primarily amorphous (Wu et al. 2018; Zhang et al. 2022a).

**Fig. 4:**
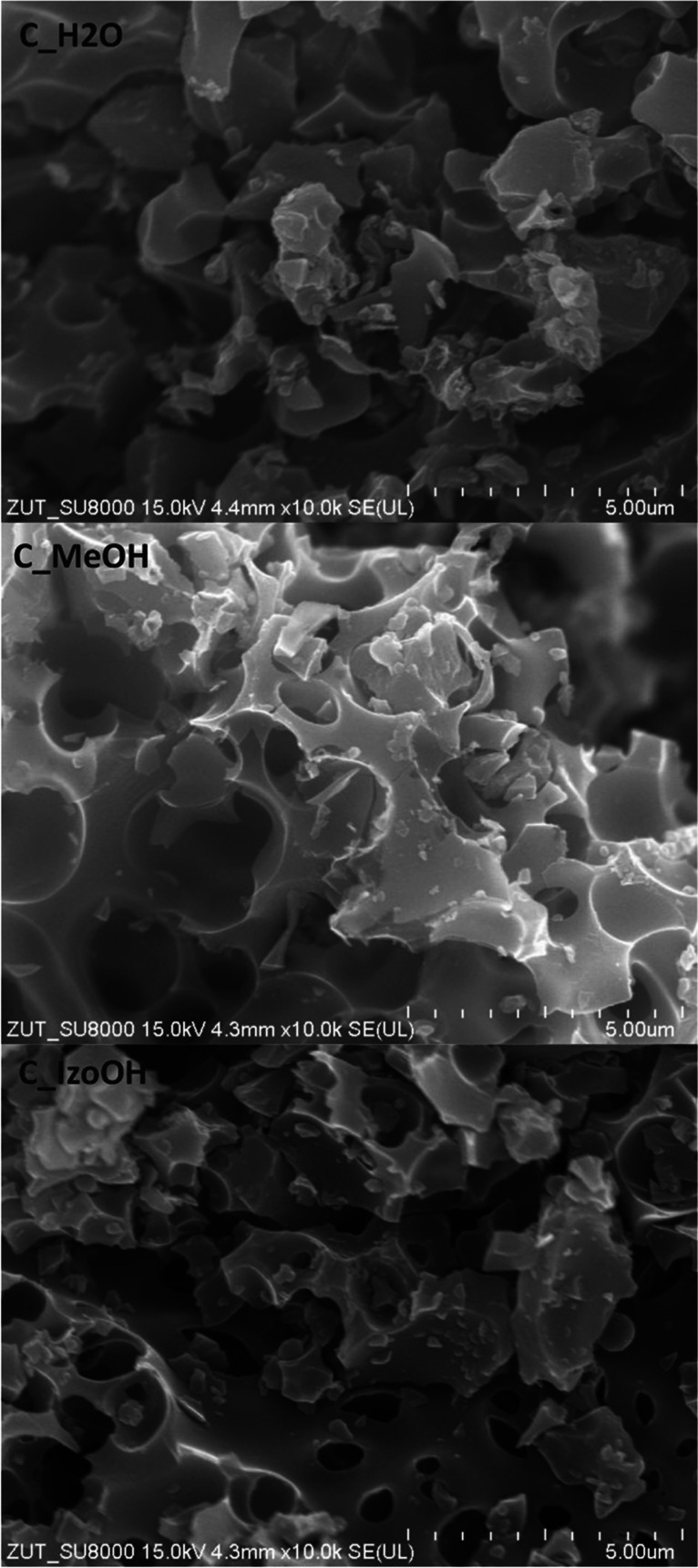
SEM images of activated carbons produced from avocado seeds using various liquids

Figure 4 shows SEM images of activated carbons produced from avocado seeds using various liquids. All the materials were similar, with many cavities on the surface of the grains as a result of potassium hydroxide etching at the temperature of 850 °C. The system of well-organized macropores was observed.

Potassium hydroxide reacted with carbon, and the gases such as CO_2_, H_2_, and H_2_O were released, making pores in carbonaceous structures (Lillo-Ródenas et al. 2003):

$$4\ \mathrm{K}\mathrm{OH}+\mathrm{C}\to 4\ \mathrm{K}+{\mathrm{CO}}_2+2\ \mathrm{\mathrm{H}}_2\mathrm{O}$$

$$6\ \mathrm{K}\mathrm{OH}+\mathrm{C}\to 2\ \mathrm{K}+3\ {\mathrm{H}}_2+2\ {\mathrm{K}}_2{\mathrm{CO}}_3$$

$$4\ \mathrm{KOH}+\mathrm{C}\to {\mathrm{K}}_2{\mathrm{CO}}_3+{\mathrm{K}}_2\mathrm{O}+2\ {\mathrm{H}}_2$$

$$6\ \mathrm{K}\mathrm{OH}+2\ \mathrm{C}\to 2\ {\mathrm{K}}_2{\mathrm{CO}}_3+2\ \mathrm{K}+3\ {\mathrm{H}}_2$$

$$4\ \mathrm{KOH}+\mathrm{C}\to {\mathrm{K}}_2{\mathrm{CO}}_3+{\mathrm{H}}_2\mathrm{O}+2{\mathrm{H}}_2+2\ \mathrm{K}$$

$$2\ \mathrm{K}\mathrm{O}\mathrm{H}+2\mathrm{C}\to 2\mathrm{C}\mathrm{O}+2\ \mathrm{K}+{\mathrm{H}}_2$$

KOH can also decompose according to:

$$2\ \mathrm{KOH}\to {\mathrm{K}}_2\mathrm{O}+{\mathrm{H}}_2\mathrm{O}$$

The produced gases can also be involved in various reactions. For example (Yang et al. 2017a):

$$\mathrm{C}+{\mathrm{H}}_2\mathrm{O}\to {\mathrm{H}}_2+\mathrm{CO}$$

$$\mathrm{CO}+{\mathrm{H}}_2\mathrm{O}\to {\mathrm{H}}_2+{\mathrm{CO}}_2$$

$${\mathrm{K}}_2\mathrm{O}+{\mathrm{CO}}_2\to {\mathrm{K}}_2{\mathrm{CO}}_3$$

$$4\mathrm{KOH}+2\mathrm{CO}\to 2{\mathrm{K}}_2\mathrm{CO}_3+2{\mathrm{H}}_2\mathrm{O}$$

$$\mathrm{C}+{\mathrm{CO}}_2\to 2\mathrm{CO}$$

$$2\ \mathrm{KOH}+{\mathrm{CO}}_2\to {\mathrm{K}}_2{\mathrm{CO}}_3+{\mathrm{H}}_2\mathrm{O}$$

$$2\ \mathrm{KOH}+\mathrm{C}+{\mathrm{H}}_2\mathrm{O}\to {\mathrm{K}}_2{\mathrm{CO}}_3+2{\mathrm{H}}_2$$

The produced potassium compounds, K_2_CO_3_, K_2_O, also could react with the char and influenced the activated carbon properties.

The textural properties allowed us to assume that activated carbons produced from avocado seed by this new method can be suitable CO_2_ sorbents. Table 2 shows the adsorption capacity of CO_2_ at a temperature of 0 °C (q_CO2_0C_) and 20 °C (q_CO2_20C_) and the adsorption capacity of N_2_ at a temperature of 20 °C (q_N2_20C_) at the pressure of 1 bar. Nitrogen adsorption was investigated in order to calculate the selectivity of CO_2_ adsorption over N_2_ in binary mixtures.

**Table 2:** The adsorption capacity of CO_2_ and N_2_ at 1 bar of activated carbons produced from avocado seeds using various liquids

AC	q_CO2_0C_	q_CO2_20C_	q_N2_20C_
	(mmol/g)	(mmol/g)	(mmol/g)
C_H2O	6.31	4.30	0.64
C_MeOH	6.47	4.13	0.59
C_IzoOH	6.47	4.35	0.55

**Fig. 5:**
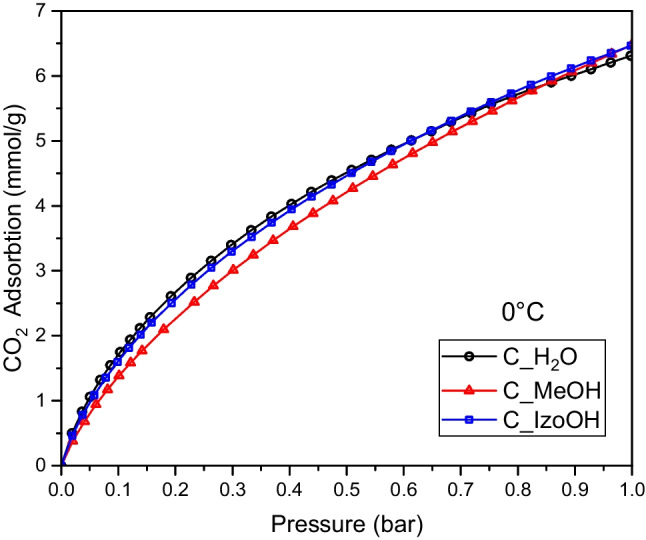
CO_2_ adsorption isotherms at a temperature of 0 °C measured for activated carbons produced from avocado seeds using various liquids. The points represent experimental data. The lines were drawn based on the Sips model

Figure 5 shows CO_2_ adsorption isotherms at the temperature of 0 °C. All the isotherms were similar. The highest CO_2_ adsorption at 1 bar and 0 °C (6.47 mmol/g) was achieved for activated carbons produced using methanol and isopropanol. When water was applied, adsorption was slightly lower, namely 6.31 mmol/g. CO_2_ adsorption at 1 bar, 0 °C on activated carbons produced from various sources is presented in Table 3. It is clearly seen that CO_2_ adsorption achieved by us using avocado seeds as a carbon source was very high compared to those presented by other researchers.

**Table 3:** CO_2_ adsorption at 1 bar, 0 °C, on activated carbons produced from various sources

Biomass	q_CO2_0C_ (mmol/g)	References
Birch	4.50	(Kishibayev et al. 2021)
Amazonian nutshells	5.13	(Serafin et al. 2021)
Lignocellulose	5.20	(Parshetti et al. 2015)
Walnut shell	5.22	(Yang et al. 2019)
Palm sheath	5.28	(Zhang et al. 2022b)
Rice husk	5.83	(He et al. 2021)
Coconut shell	6.04	(Yang et al. 2017b)
Andiroba shells	6.10	(Serafin et al. 2022)
Hazelnut shell	6.44	(Ma et al. 2022)
Avocado seeds	6.47	This work

The kinetic diameter of CO_2_ molecules is 0.33 nm (D’Alessandro et al. 2010). The pores about two times larger than 0.33 nm are most favorable for CO_2_ adsorption. The adsorption potential affected by CO_2_ molecules from opposite walls in such micropores is the highest (Ghimire et al. 2019). All the samples were ultramicroporous, so the CO_2_ adsorption was so high.

**Fig. 6:**
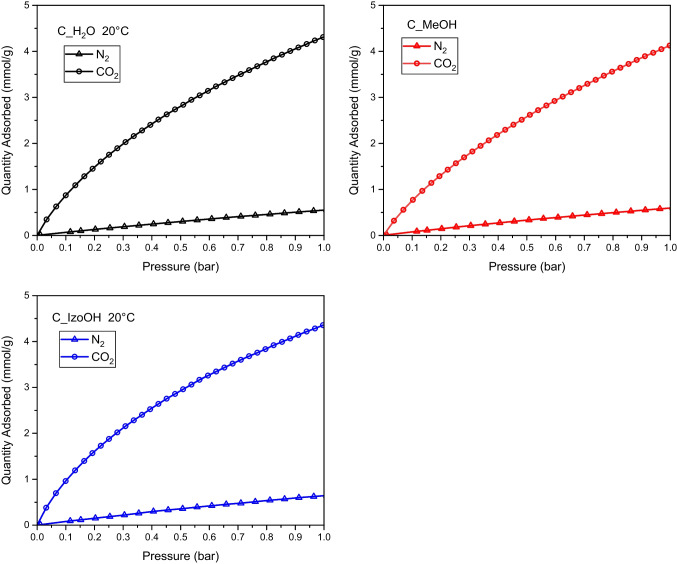
CO_2_ and N_2_ adsorption isotherms at a temperature of 20 °C measured for activated carbons produced from avocado seeds using various liquids. The points represent experimental data. The lines were drawn based on the Sips model

Figure 6 presents CO_2_ and N_2_ adsorption isotherms at a temperature of 20 °C. The values of the CO_2_ adsorption at higher temperatures were lower but still high. The highest CO_2_ adsorption at 1 bar exhibited C_IzoOH (4.35 mmol/g), and the lowest adsorption (4.13 mmol/g) was observed over C_MeOH. The values of CO_2_ adsorption at 20 °C and 1 bar were strongly connected with microporosity, namely V_tot/_V_micro_ values. As was proved by others (Wickramaratne and Jaroniec 2013), CO_2_ adsorption on activated carbons at ambient conditions is strongly dependent on microporosity (Table 1). The decrease of the CO_2_ adsorption with the increase of the temperature indicated that physical adsorption took place.

Two two-parameter models (Freundlich and Langmuir) and two three-parameter models (Toth and Sips) were applied to analyze the experimental adsorption isotherms. The Freundlich , Langmuir, Toth, and Sips equations were described in Ayawei et al. (2017) and Serafin et al. (2023).

The applicability of the equations to fit the experimental data was established using the least-squares method. The lowest values of the errors was obtained using Sips model for CO_2_ and N_2_ adsorption. The Sips model is given by the equation:

$$q=\frac{q_m\bullet b\bullet {p}^n}{1+b\bullet {p}^n}$$

where

*q*: The gas equilibrium adsorption at pressure *p*
*p*: Equilibrium pressure
*q*_*m*_: The saturation capacity
*b*: Equilibrium constant
*n*: Exponential parameter representing the heterogeneity of the material

The obtained parameters are presented in Table 4.

**Table 4:** The Sips model parameters and standard error calculated based on experimental data of CO_2_ and N_2_ adsorption

AC	Temp.	*q*_*m*_	*b*	*n*	Error
	[oC}	[mmol/g]	[/bar]		
		Carbon dioxide			
C_H2O	0	14.86	0.74	0.76	3.16$\cdot$10^-03^
	20	14.05	0.44	0.82	1.66$\cdot$10^-03^
C_MeOH	0	26.35	0.33	0.77	2.40$\cdot$10^-03^
	20	17.61	0.31	0.83	6.78$\cdot$10^-04^
C_IzoOH	0	18.89	0.52	0.75	3.44$\cdot$10^-03^
	20	12.28	0.55	0.81	6.27$\cdot$10^-04^
		Nitrogen			
C_H2O	20	3.53	0.19	0.98	1.13$\cdot$10^-05^
C_MeOH	20	4.12	0.17	0.95	1.80$\cdot$10^-05^
C_IzoOH	20	3.11	0.26	1.00	1.21$\cdot$10^-04^

The saturation capacity decreased with the temperature increase, which confirmed the physical adsorption. The exponential parameters were close to one, proving the surface’s homogeneity. The values calculated by Sips equations are presented in Figs. 5 and 6 as lines, while points represented experimental data.

The N_2_ adsorption measurements were performed to calculate the CO_2_ adsorption selectivity over N_2_. The ideal adsorbed solution theory (IAST) (Myers and Prausnitz 1965) was applied to calculate the selectivity of carbon dioxide over nitrogen at 20^o^C. The selectivity of g1 over g2 is possible to calculate based on single adsorption isotherms of g1 and g1:

$${S}_{(g1)}=\frac{\frac{x_{g1}}{y_{g1}}}{\frac{x_{g2}}{y_{\boldsymbol{g}\textbf{2}}}}$$

where

x_g1_ (x_g2_): The molar fractions of g1 (g2) gas in the adsorbed phase
y_g1_, (y_g2_): The molar fractions of g1 (g2) gas in the bulk phase

The accuracy of the IAST calculation was established for many gas mixtures on various sorbents (Herm et al. 2011; Lu et al. 2011).

Based on the IAST theory, the selectivity of CO_2_ adsorption for the equimolar mixture was calculated according to the equation:

$${S}_{CO_2}=\frac{q_{CO_2(p)}}{q_{N_2(p)}}$$

The sorbents may be applied for CO_2_ removal from flue gas. The content of CO_2_ in off-gas depends on the kind of fossil fuels. If fuel gas is the energy source, the CO_2_ concentration is about 10%. For coal burning, CO_2_ concentration is equal to 15%. Taking into account the CO_2_ content in flue gas, the selectivity of CO_2_ over N_2_ for 10% and 15% CO_2_ content was also calculated.

$${S}_{\left({CO}_2\_10\ or\ 15\right)}=\frac{q_{CO_2\left({p}_{CO2}\right)}}{p_{CO_2}}:\frac{q_{N_2\left({p}_{N2}\right)}}{p_{N_2}}$$

*q*_*i*_(*p*): Adsorption value of i at pressure pi

For carbon dioxide, p_CO2_ were equal 0.1 and 0.15. For nitrogen, p_N2_ were equal to 0.9 and 0.85. In order to establish CO_2_ and N_2_ adsorption at a given pressure, the Sips model was applied.

**Fig. 7:**
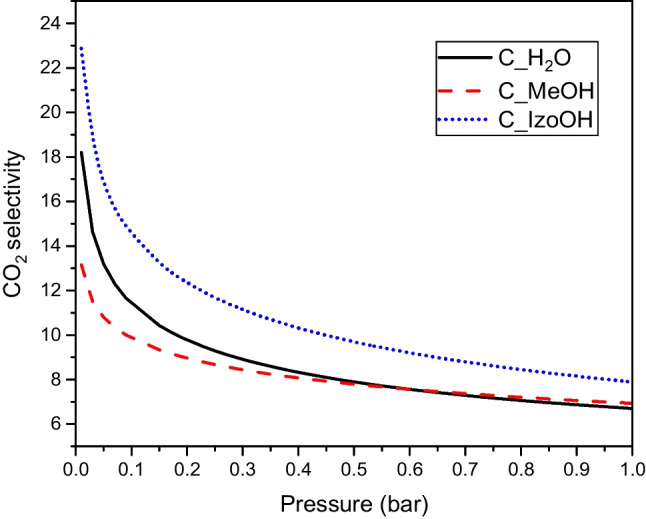
The selectivity of CO_2_ adsorption for the equimolar mixture of carbon dioxide and nitrogen at a temperature of 20 °C

The selectivity of CO_2_ adsorption for the equimolar mixture are presented in Fig. 7.

The highest CO_2_ adsorption was achieved over C_IzoOH activated carbon. Selectivity decreased with increasing pressure. The course of the curves was typical (Kiełbasa et al. 2022).

The selectivities of CO_2_ adsorption over N_2_ for typical flue gas concentrations 10 and 15% (S_CO2_10,_ S_CO2_15_) are presented in Table 5. The highest selectivity was observed for C_IzoOH.

**Table 5:** The selectivity of CO_2_ adsorption over N_2_ for typical flue gas concentration 10 and 15% (SCO2_10, SCO2_15)

AC	S_CO2_10_	S_CO2_15_
C_H2O	13	12
C_MeOH	12	11
C_IzoOH	17	15

**Fig. 8:**
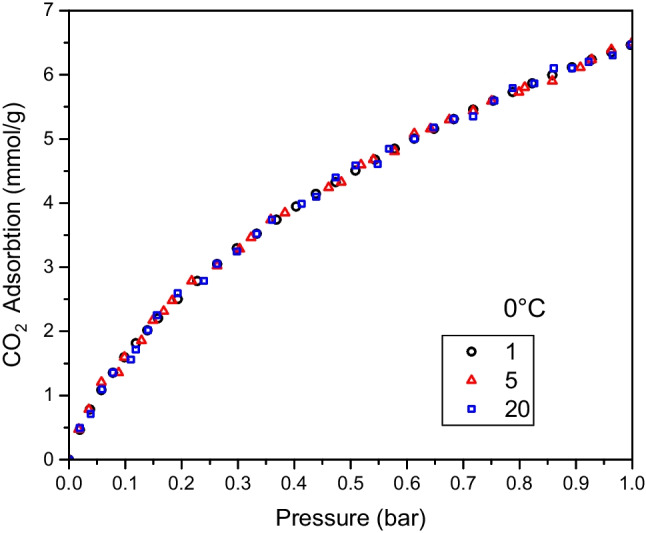
Adsorption multicyclic stability tests at 0 °C for the C_Izo sample after 1, 5, and 20 cycles

The reusability measurements of the best sorbent, C_Izo, are performed and presented in Fig. 8. Twenty CO_2_ adsorption/desorption cycles were performed at 0 °C and up to 1 bar for the best sorbent (C_Izo). As shown in Fig. 8, the CO_2_ adsorption capacity of this activated carbon was well maintained even after 20 cycles of adsorption/desorption processes. Intermediate cycles are not shown in the figure for clarity.

The Gibbs free energy change for the CO_2_ adsorption over C_Izo sample was calculated according to the equation (Tiwari et al. 2017):

$$\Delta {G}^o=- RTLn(K)$$

where K is the equilibrium constant for CO_2_ adsorption

ΔH^o^ and ΔS^o^ can be obtained from the slope and intercept of van’t Hoff plot (Goel et al. 2015)

The negative values of Gibbs free energy change were obtained: − 1.03kJ/mol and − 0.56 kJ/mol for the temperature of 0 and 20 °C, respectively. Negative values indicated the feasibility and spontaneity of the adsorption process. Very small values of enthalpy and entropy were obtained: 0.08 J/mol and 0.03 kJ/(mol K). The negative value of enthalpy confirmed the exothermic nature of the adsorption. The positive value of entropy demonstrated the increase in randomness at the adsorbent-adsorbate interface and also adsorbent’s affinity towards carbon dioxide. Small value of ΔS^o^ indicated that no signicant change in entropy took place.

## Conclusions

In summary, we synthesized ultramicropore activated carbons from avocado seeds using combined solvothermal carbonization and thermal KOH activation method. The activated carbons with excellent textural properties (pore volume, micropore volume, and specific surface area) were successfully synthesized. All the samples were very good CO_2_ sorbents. The micropores were very important for CO_2_ adsorption. The highest CO_2_ adsorption and CO_2_ selectivity over N_2_ were achieved for activated carbon obtained using isopropanol that exhibited the highest micropore percentage making this material a promising candidate for carbon dioxide removal from mixtures of gasses. The highest CO_2_ adsorption at a pressure of 1 bar was achieved for C_Izo was equal to 6.47 mmol/g at 0 °C and 4.35 mmol/g at 20 °C. Because of the high CO_2_ adsorption, CO_2_ selectivity, and stability, this material can be applied for CO_2_ capture from flue gas.

## Data Availability

Not applicable.
